# Application of hydrostatic CCC–TLC–HPLC–ESI-TOF-MS for the bioguided fractionation of anticholinesterase alkaloids from *Argemone mexicana* L. roots

**DOI:** 10.1007/s00216-015-8468-x

**Published:** 2015-01-25

**Authors:** Wirginia Kukula-Koch, Tomasz Mroczek

**Affiliations:** Department of Pharmacognosy with Medicinal Plant Unit, Medical University of Lublin, 1 Chodzki St., 20-093 Lublin, Poland

**Keywords:** *Argemone mexicana*, Papaveraceae, TLC–HPLC–MS, Hydrostatic counter-current chromatography, Galanthamine, Isoquinoline alkaloids

## Abstract

A rapid hydrostatic counter-current chromatography–thin-layer chromatography–electrospray-ionization time-of-flight mass spectrometry (CCC–TLC–ESI-TOF-MS) technique was established for use in seeking potent anti-Alzheimer’s drugs among the acethylcholinesterase inhibitors in *Argemone mexicana* L. underground parts, with no need to isolate components in pure form. The dichloromethane extract from the roots of Mexican prickly poppy that was most rich in secondary metabolites was subjected to hydrostatic-CCC-based fractionation in descending mode, using a biphasic system composed of petroleum ether–ethyl acetate–methanol–water at the ratio of 1.5:3:2.1:2 (*v*/*v*). The obtained fractions were analyzed in a TLC-based AChE-inhibition “Fast Blue B” test. All active components in the fractions, including berberine, protopine, chelerithrine, sanguinarine, coptisine, palmatine, magnoflorine, and galanthamine, were identified in a direct TLC–HPLC–ESI-TOF-MS assay with high accuracy. This is the first time galanthamine has been reported in the extract of Mexican prickly poppy and the first time it has been identified in any member of the Papaveraceae family, in the significant quantity of 0.77 %.

**Graphical Abstract:**
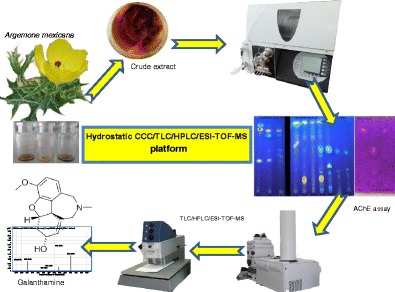
Screening platform of anti-AChE alkaloids

Screening platform of anti-AChE alkaloids

## Introduction

Neurodegeneration-related diseases are a growing health problem in aging populations worldwide. It is estimated that the number of patients suffering from Alzheimer’s disease (AD) alone is more than 25 million and that the incidence will double by 2050 [1]. Plant-derived secondary metabolites have been revealed to be interesting sources of potent drugs with procognitive properties. This is the case for galanthamine, an alkaloid from plants of the Amaryllidaceae family, and for huperzine A, produced by lycopods. Galanthamine is a registered drug for treating Alzheimer’s and Huntington’s diseases, and huperzine A is sold as a procognitive food supplement [2].

These compounds are characterized by the ability to reduce the activity of acetylcholinesterase (AChE), which, by decomposing acetylcholine in the synaptic cleft, impedes neurotransmission and thus causes cognition impairment. The inhibition of this enzyme, which is targeted by different medicines, guarantees a protective function against dementia [3].

Although a variety of therapeutic strategies believed to delay neurodegeneration processes have been described, there is a strong need to develop new medicines characterized by a better pharmacological profile or reduced side effects.

This study presents a rapid screening procedure enabling the identification of natural anti-AChE products present in mixtures, with no need to isolate the products. After hydrostatic-counter-current-chromatography (CCC)-based fractionation, the thin-layer chromatography–mass spectrometry (TLC–MS) interface used extended into a two-dimensional (2D) TLC–high-powered (HP) LC–electrospray-ionization time-of-flight (ESI-TOF)-MS system provides direct MS analysis of any spot obtained after the TLC development, including more complicated bands produced after bioanalysis.

The authors chose *Argemone mexicana* L. (Mexican poppy) for the evaluation of this method, because use of the TLC–HPLC–MS method has never been described for any Papaveraceae plant. *A. mexicana* is a spiny, bitter annual plant growing abundantly in the wastelands of Mexico and western South America. The plant is widely naturalized in countries with a similar climate, including Brazil, India, Ethiopia, and Bangladesh [4, 5]. Several protoberberine, benzophenanthridine, benzylisoquinoline, and protopine alkaloids have been documented in Mexican-prickly-poppy extracts, and are linked with the anti-inflammatory, hypoglycaemic, and antihypertensive properties of the plant [6–9]. The broad distribution of the species and its rich alkaloid composition explain the authors’ decision to attempt successful and universal optimization of a TLC-based identification of active components. Moreover, total extracts from the Mexican poppy have been previously reported to be active against AChE, in a screening study performed on the enzyme isolated from human erythrocytes (RBC) by Srivastava et al. [10]. The recognition of active components from *A. mexicana* is therefore of the greatest necessity.

Rough fractionation of the dichloromethane (DCM) extract from *A. mexicana* roots was performed by means of CCC. The principle of separation involves the partition of a solute between two immiscible solvents in a continuous distribution, which eliminates both complications typical for alkaloids, involving irreversible adsorptive sample loss, and temperature-affected deactivation or tailing of solute peaks [11, 12]. The recovery of products is approximately 100 % for CCC, and solvent consumption is low. In addition, the system has high selectivity, high peak resolution, and large injection volume, making this method favorable in comparison to the traditionally used HPLC [13].

In this study, a CCC-based fractionation was followed by TLC-bioautography-based “Fast Blue B reagent” anticholinesterase activity assay. In addition, the composition of the obtained fractions was investigated by TLC–HPLC–ESI-TOF-MS analysis. The identification of active compounds was on the basis of their fragmentation pattern, literature data, and respective standards comparison for both MS and TLC.

## Materials and methods

### General experimental procedures

The standards of galanthamine hydrobromide, berberine hydrochloride, sanguinarine hydrochloride, and chelerithrine hydrochloride were purchased from Sigma–Aldrich (St. Louis, MO, USA), as were acetylcholinesterase from *Electrophorus electricus* (lyophilized powder type VI-S, 200–600 units per mg protein), 2-naphthyl acetate, and Fast Blue B Salt.

CCC separation was performed using an Armen SCPC-250-L system (Saint Ave, France) with a ternary high-pressure gradient pump and UV detector. Gradient-grade solvents used to prepare solvent systems for CCC separation came from The Polish Reagents (POCH, Gliwice, Poland).

HP-TLC silica-gel-coated plates F_254_ (10 × 20 cm) and Dragendorff reagent were obtained from Merck (Darmstadt, Germany). Methanol, acetonitrile, ammonia, and formic acid of HPLC grade were produced by J.T. Baker (Gross-Gerau, Germany), as were methanol and water for spectrometry use.

A Zorbax RP 18 Stable Bond (150 × 2.1 mm, *d*_p_ = 3.5 μm) (Agilent Technologies) HPLC column was used in chromatography separation for LC–MS applications.

An Agilent Technologies Series 1200 HPLC/PDA system was used for the preparation of CCC systems. TLC–HPLC–ESI-TOF-MS analysis was performed using the Agilent G3250AA LC/6210 MSD TOF system containing a TLC–MS interface (Camag) connected via a Zorbax RP-18 Rapid Resolution 50 × 2.1 mm, *d*_p_ = 5 μm column compartment of an HP 1200 chromatograph equipped with a binary pump, a column thermostat, an autosampler, and a photodiode-array detector and LC/6210 MSD spectrometer (Agilent Technologies, Santa Clara, CA, USA) with ESI dual-spray source (for sample and reference-mass solutions).

### Plant material

Dried roots of *Argemone mexicana* L. were obtained from the garden of the Chair and Department of Pharmacognosy with Medicinal Plant Unit, Medical University of Lublin, and were authenticated by the authors and Dr Michał Hajnos. A voucher specimen (WK1010001) was deposited in the same department.

### Extraction and isolation

#### Extraction

Roots of *Argemone mexicana* L. (156 g) were pulverized into a coarse-grained powder and macerated three times using dichloromethane, methanol, and water, respectively. Evaporation of the obtained extracts was performed under reduced pressure and yielded 5.23, 4.81, and 9.14 g crude extract, respectively. TLC extract profiling revealed the highest diversity of secondary metabolites to be in the dichloromethane extract, and this extract was therefore separated by means of CCC chromatography.

#### Biphasic-solvent-system selection

The choice of a separation mixture was made on the basis of existing research data and Sorensen diagrams and was validated by TLC and HPLC methods.

Separation of the extract constituents between upper and lower phases was performed in 16 chosen solvent systems (see Table 1). Each solvent mixture (5 mL) was prepared in 10 mL vials. Extract (2 mg) was vortex mixed in every system and left for separation of the phases. The settling time of a sample was measured. The samples which produced emulsion were rejected at this stage. Several TLC plates of upper and lower phases were developed in methanol–dichloromethane (10:90) (*v*/*v*) with addition of 5 % ammonia, to assess the degree of metabolite partitioning between two phases.

**Table 1:** Biphasic solvent systems prepared for the evaluation of the CCC separation method on dichloromethane extract from *Argemone mexicana* L. roots

No.	Solvent system	Ratio (*v*/*v*)	Remarks	Ref.
1	CH_2_Cl_2_–MeOH–H_2_O	4.8:1.6:3.6	Rejected	[14]
2	AcOEt–MeOH–H_2_O	5:1:4	Rejected	[14]
3	BuOH–MeOH–H_2_O	4.6:1.0:4.4	Rejected	[14]
4	*n*-Hexane–AcOEt–MeOH–H_2_O	2:5:2:5	Rejected	[15]
5	CHCl_3_–MeOH–0.2 mol L^−1^ HCl	4:1.5:2	LC evaluation	[16]
6	Petroleum ether–AcOEt–MeOH–H_2_O	1.5:3.0:2.1:2.0	LC evaluation	[16]
7	BuOH–*c*-hexane–EtOH–H_2_O	8:2:5:10	Rejected	Sorensen diagrams
8	BuOH–*c*-hexane–ACN–H_2_O–0.3 % HCl	8:2:5:13	Rejected	Sorensen diagrams
9	Heptane–AcOEt–MeOH–H_2_O	1:4:1:4	Rejected	[17]
10	Heptane–AcOEt–MeOH–H_2_O	1:1:1:1	LC evaluation	[17]
11	Heptane–AcOEt–MeOH–H_2_O	3:1:3:1	Rejected	[17]
12	Heptane–AcOEt–MeOH–H_2_O	1:0:1:0	Rejected	[17]
13	Diethyl ether–AcOEt–MeOH–H_2_O	1:2.1:1.5:1.4	Rejected	Sorensen diagrams
14	Diethyl ether–AcOEt–ACN–H_2_O	1:2.1:1.5:1.4	LC evaluation	Sorensen diagrams
15	Diethyl ether–AcOEt–MeOH–H_2_O	1:2.1:1.5:1	Rejected	Sorensen diagrams
16	Diethyl ether–AcOEt–MeOH–0.3 % HCl	1:2.1:1.5:1.4	Rejected	Sorensen diagrams

#### K-value evaluation by HPLC–ESI-TOF-MS

HPLC–DAD–ESI-TOF-MS analysis was performed for all solvent systems selected for LC evaluation.

As a mobile phase, the following gradient of solvent mixture A (MeCN and 0.1 % formic acid) in B (water and 0.1 % formic acid) was used: 0–10 min linear gradient of A from 10 to 40 %, 10–12 min isocratic flow of 40 %, 12–17 min gradient from 40 to 95 %, 17–20 min quick gradient from 95 to 10 %, and finally 20–22 min isocratic run with 10 % A in B. The total analysis time was set at 22 min, the flow at 0.2 mL min^−1^, the post time at 4 min, injection volume at 5 μL, and thermostat temperature at 25 °C. UV detection was performed at 260 and 280 nm. A Zorbax RP-18 Stable Bond (150 × 2.1 mm, *d*_p_ = 3.5 μm; Agilent Technologies) HPLC column was used in chromatography separation.

The ESI-TOF-MS system was optimized in the positive mode regarding sensitivity, resolution, and mass accuracy. The resolution of the TOF-MS instrument was set after careful tuning of the instrument. Typically, it was higher than 12,000 for the highest peak of the calibration mixture at *m*/*z* 2721.894829. A set of 10 different accurate masses of the tuning and calibration mixture was used both for tuning and mass calibration. The whole process of tuning and calibration regarding ion abundances, resolution, and high mass accuracy is described elsewhere [18].

After the tuning and the calibration procedures, the detailed MS settings were: gas temperature: 350 °C, vaporizer temperature: 350 °C, drying-gas flow: 10 L min^−1^, nebulizer: 30 psig, fragmentor voltage: 175 or 225 V, skimmer voltage: 65 V, capillary voltage: 2000 V, and ionization source: ESI. All experiments were conducted in the positive mode with the addition of internal standards (MW: 121.0508 and MW: 922.0097). The mass spectra were recorded in a mass range 150 to 1000 *m*/*z*. Mass Hunter Workstation software (version B.02.00) was used in the data acquisition and analysis.

Partition-coefficient values (*K*) were calculated for all major peaks in the extracts by the equation: *K* = (peak area in the upper phase)/(peak area in the lower phase).

#### Counter-current separation of dichloromethane root extract

The sample (377 mg dichloromethane extract) was dissolved in 6 mL biphasic solvent system (1:1) and injected, after equilibration at 500 rpm, 20 mL min^−1^ for 15 min, at a constant rotation speed of 1800 rpm. Flow throughout the run was 8 mL min^−1^ in normal-phase mode (lower phase stationary). Elution-extrusion operation mode obtained 18 fractions, collected within 42 min. Then a further 18 fractions were eluted with stationary phase within a further 44 min with fresh stationary phase, still in the ascending mode.

The composition of all fractions was evaluated by TLC, and then all fractions were mixed in accordance with their quantitative composition. Every combined fraction was submitted to bioassay.

#### Thin-layer chromatography of the fractions

Fractions from CCC separation were analyzed by TLC. Plates were cut in half (10 × 20 cm) and carefully spotted in evenly spaced dashes using Camag Autosampler System (Muttenz, Switzerland). Samples were applied to the plates as 5 mm spots or bands, 10 mm apart, 15 mm from the left and lower edges. The development was performed in Camag unsaturated glass TLC chambers, using the solvent system methanol–dichloromethane (10:90) (*v*/*v*) with the addition of 5 % ammonia, in 64 % humidity. Dried TLC were examined under UV light at 254 and 365 nm and derivatized with Dragendorff reagent (Merck, Darmstadt, Germany) until vivid orange spots of alkaloids became visible. Similar fractions were mixed and evaporated to dryness. The combined fractions were developed on HP-TLC plates in the same solvent system. Four were prepared simultaneously to be sprayed with Dragendorff and vanillin reagents, to be analyzed by TLC–HPLC–MS, and to be subjected to the anticholinesterase test.

### TLC-bioautography towards AChE inhibition

The modified “Fast Blue B reagent” method was as described by Mroczek [18]. A solution of all combined fractions from CCC separation (10 μL, 0.2 mg per 10 mL) and of 10 μL 0.29 mg per 10 mL solution of galanthamine standard (Sigma–Aldrich) were spotted on a TLC using the Camag autosampler. HP-TLC silica gel 60 F254 aluminium sheets were developed in the mobile phase dichloromethane–methanol (9:1, *v*/*v*) with the addition of 25 % ammonia (0.5 mL ammonia per 50 mL mobile phase) and 2.2 mmol L^−1^ naphthyl 2-acetate (30 mg per 20 mL mobile phase). Subsequently they were dried at room temperature and sprayed as follows:

1. AChE (3 U mL^−1^ in Tris buffer pH 7.8, stabilized with bovine serum) incubated for 20 min at 37 °C; and
2. Fast Blue B Saltwater solution (0.615 mg mL^−1^) visualized after approximately 1 min.

### TLC–HPLC–ESI-TOF-MS analysis

TLC–HPLC–ESI-TOF-MS analysis was performed first for the major compounds present in the purified fractions, and later for the spots which decomposed the AChE enzyme on unsprayed HP-TLC plates. An isocratic system composed of a mixture of A (MeCN and 1 % formic acid) in B (water and 1 % formic acid) was used as the mobile phase, in the ratio of 50:50 (*v*/*v*).

A TLC–MS interface (Camag) was used to collect the spots’ content, and they were then analyzed using a spectrometer. The total analysis time was set at 5 min for each spot, and the application time of the interface was 3 s.

A short Zorbax RP-18 Rapid Resolution 50 × 2.1 mm, *d*_p_ = 5 μm column was mounted to separate the impurities extracted directly from the TLC plates. The detailed MS settings were similar to those presented in the section “*K*-value evaluation by HPLC–ESI-TOF-MS”.

## Results and discussion

### Counter-current separation of dichloromethane extract

**Fig. 1:**
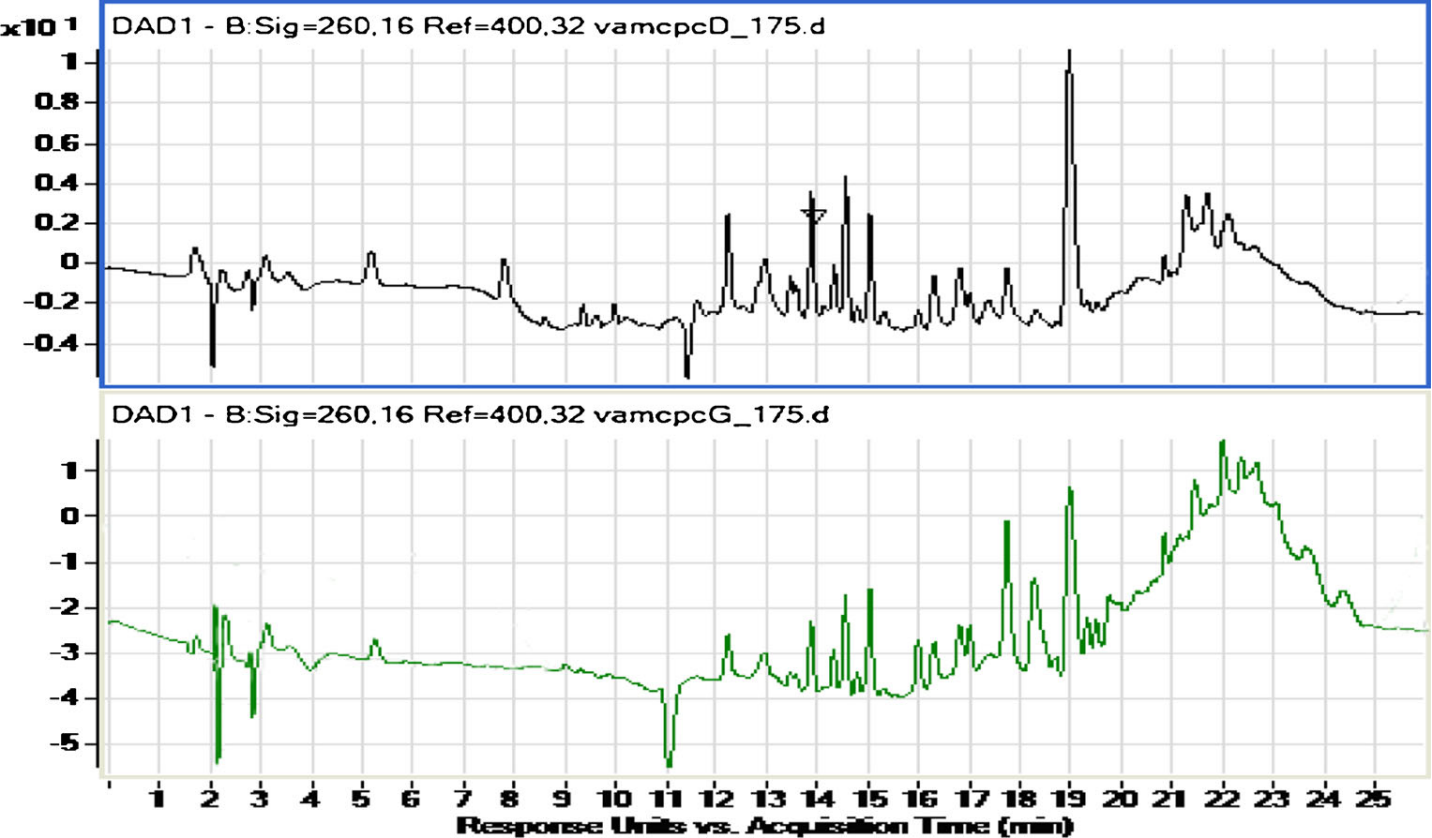
Composition of lower (*top chromatogram*) and upper (*bottom chromatogram*) phases in LC chromatograms recorded for fraction 6 (Table 1) at 260 nm

In the hydrostatic-CCC separation of the DCM extract, the choice of a suitable isolation mixture was on the basis of favorable partition-coefficient values in a biphasic solvent system. The partition coefficient (*K*), defined as the mass-concentration ratio of a given compound in the lower phase versus its content in the upper phase [19], should remain within the range of 0.67–1.5. After consulting the literature and Sorensen diagrams and modifying the presented biphasic-solvent compositions, the most suitable solvent systems were selected for LC injection and *K*-value calculations. Solvent system 6 (Table 1) was chosen as the best for the separation of the investigated extract (Fig. 1). It consisted of four different solvents (petroleum ether–ethyl acetate–methanol–water) at the ratio of 1.5:3:2.1:2 (*v*/*v*). The *K* values of the major compounds ranged from 0.6 to 1.8, resulting in a global *K* value of 0.89. On the basis of these observations, an aqueous phase was chosen as stationary phase and the fractionation was performed in the ascending mode. The composition of the upper and lower phases of the best biphasic system is presented in Fig. 1.

### TLC–HPLC–ESI-TOF-MS analysis of extract

**Fig. 2:**
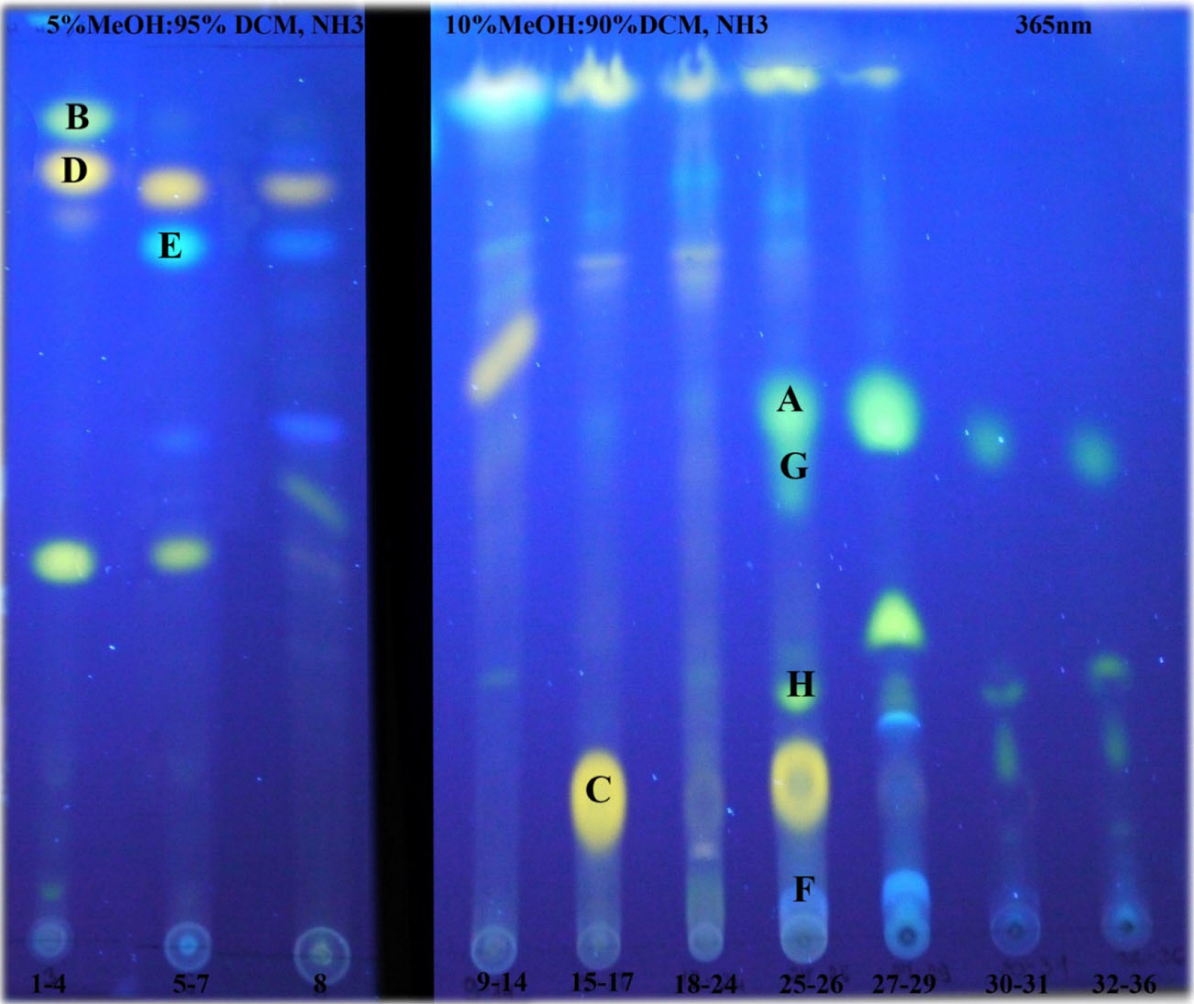
Major fractions obtained after CCC separation in UV *λ* = 365 nm (*A*, berberine; *B*, chelerithrine; *C*, protopine; *D*, sanguinarine; *E*, galanthamine; *F*, magnoflorine; *G*, palmatine; *H*, coptisine)

The DCM extract afforded 10 main fractions, which were successfully separated by HP-TLC in two solvent systems: MeOH–DCM–NH_3_ 5:90:5 (*v*/*v*) and 10:85:5 (*v*/*v*) (Fig. 2). Major spots were very efficiently analyzed by the TLC–HPLC–MS system, leading to the rapid identification of several bioactive alkaloids (Table 2), including berberine, chelerithrine, coptisine, protopine, sanguinarine, galanthamine, magnoflorine, and palmatine. Moreover, the TLC–HPLC–MS system enabled the identification of protopine, palmatine, magnoflorine, and coptisine with no need of reference solutions.

**Table 2:** TLC–HPLC–ESI-TOF-MS accurate mass measurements of alkaloids identified in the DCM extract of *Argemone mexicana* L. roots

Compound	Molecular formula	*m*/*z* exp.	*m*/*z* calc.	Delta (ppm)	RDB	In-source ESI-TOF-MS fragments	Compound	Ref.
A	C_20_H_18_NO_4_	335.1155	335.1158	0.7	13	321 [M − 15]^+^ [M − CH_3_]^+^ 306 [M − 2 × 15]^+^ [M − 2 × CH_3_]^+^ 292 [M − 2 × 15 − 14]^+^ [M − 2 × CH_3_ − CH_2_]^+^	Berberine	[20, 21]
B	C_21_H_17_NO_4_	348.1161	348.1158	−1.05	14	333 [M − 15]^+^ [M − CH_3_]^+^ 318 [M − 2 × 15]^+^ [M − 2 × CH_3_]^+^ 304 [M − 44]^+^ [M − CO_2_]^+^	Chelerithrine	[22]
C	C_20_H_19_NO_5_	353.1259	353.1263	1.13	12	338 [M − 16]^+^ [M − O]^+^ 336 [M − 18]^+^ [M − H_2_O]^+^ 323 [M − 16 − 15]^+^ [M − O − CH_3_]^+^	Protopine	[22, 23]
D	C_20_H_13_NO_4_	332.0920	332.0845	−0.8	15	317 [M − 15]^+^ [M − CH_3_]^+^ 302 [M − 2 × 15]^+^ [M − 2 × CH_3_]^+^ 289 [M − 74]^+^ [M − 2 × CH_3_ − CO_2_]^+^ 274 [M − 58]^+^ [M − CH_2_O + CO]^+^	Sanguinarine	[22]
E	C_17_H_21_NO_3_	287.1593	287.1521	0.42	8	310 [M + 23]^+^ [M + Na]^+^ 270 [M − 18]^+^ [M − H_2_O]^+^ 261 [M − 28]^+^ [M − CO]^+^ 231 [M − 57]^+^ [M − H − CH_2_CHNHCH_3_]^+^ 213 [M − 18 − 57]^+^ [M − H_2_O − HCH_2_CHNHCH_3_]^+^	Galanthamine	[24]
F	C_20_H_23_NO_4_	342.1632	342.1627	−1.5	10	312 [M − 2 × 15]^+^ [M − 2 × CH_3_]^+^ 297 [M − 3 × 15]^+^ [M − 3 × CH_3_]^+^ 282 [M − 4 × 15]^+^ [M − 4 × CH_3_]^+^	Magnoflorine	[20]
G	C_21_H_22_NO_4_	352.1527	352.1554	−1.5	12	337 [M − 15]^+^ [M − CH_3_]^+^ 322 [M − 2 × 15]^+^ [M − 2 × CH_3_]^+^ 308 [M − 3 × 15]^+^ [M − 3 × CH_3_]^+^ 293 [M − 4 × 15]^+^ [M − 4 × CH_3_]^+^	Palmatine	[20–22]
H	C_19_H_14_NO_4_	320.0917	320.0928	−0.6	14	321 [M]^+^	Coptisine	[20, 22]

High-resolution (HR) mass spectrometry has an important function in the analysis of different types of chemical structure of a variety of metabolites of plant origin [18, 20, 21, 24, 25]. It can be hyphenated with chromatographic systems, e.g. HPLC–TOF-MS, HPLC–quadrupole (Q)-TOF-MS, or HPLC–Orbitrap [23–25]. We used an ESI-TOF-MS instrument, quite an inexpensive system compared with the hybrid Q-TOF or Orbitrap analyzers. The system used made it possible to analyze the elemental composition of the organic molecules and their corresponding product ions. For all investigated compounds, HR mass spectra with high mass accuracy (error was recorded at 0–2 ppm levels) enabled molecular-formula determination, with no doubt regarding a given number of defined elements (C, H, N, and O, the presence of which was analyzed in the identified alkaloids) (Table 2). All experiments were recorded in the positive mode.

On the basis of an in-source compound decomposition, the HR fragmentation pathways were studied. This enabled the determination of seven different inhibitors belonging to the isoquinoline type of alkaloids, and of one amaryllidaceae alkaloid, galanthamine. The ESI-TOF-MS fragmentation of galanthamine was in agreement with our work described elsewhere [24]. For the isoquinoline alkaloids, the following fragment ions were typically observed: demethylation [M − 15], deethylation [M − 29], deoxidation [M − 16], demethoxylation [M − 31], dehydration [M − 18], decarboxylation [M − 44], and decarbonylation [M − 28] ions, which is in accordance with literature data and reference substances. The presence of the isoquinoline alkaloids identified by ESI-TOF-MS had already been reported.

The structures of all released moieties were established beyond doubt, on the basis of HR ESI-TOF-MS spectra and of ChemStation software’s unequivocal suggestions. The mass-spectrometry data of the investigated compounds were compared with literature data for the determination (see Table 2). All the compounds were known ones, so no complementary NMR studies were required.

The introduction of a short chromatographic column between the TLC interface and ESI source increased the purity of the spectra. As a result, the major components were recorded in the mass spectra as purified from other tailing compounds. Details of the fragmentation, accurate molecular masses, double-bond equivalent values (RDB), and molecular formulae of the compounds are presented in Table 2.

TLC–HPLC–MS provided analytical data for a rapid structure elucidation of this mixture of compounds. Moreover, high loadings of the material on the TLC plate enabled easier identification of compounds, including those present in the mixture in smaller quantities. Despite the expected lower reproducibility for TLC, the reproducibility of retention-factor (Rf) values and mass spectra was excellent.

This approach is new for isoquinoline alkaloids. Many research papers have combined TLC chromatography with MS, but only using preparative TLC plates as isolation tools in an offline procedure. The approach presented in this paper does not include the previously necessary and time-consuming isolation, but only rough fractionation of the mixtures, which is sufficient to produce pure spots on a TLC plate and enable their identification by MS. This technique can be used for different groups of secondary metabolites other than alkaloids, in a variety of TLC solvent systems, and for assessing different bioactivities, which makes this approach very universal. The traditional Rf-based identification, including the comparison with a standard, could be substituted with a modern direct-MS-based identification with no need to purchase reference compounds.

**Fig. 3:**
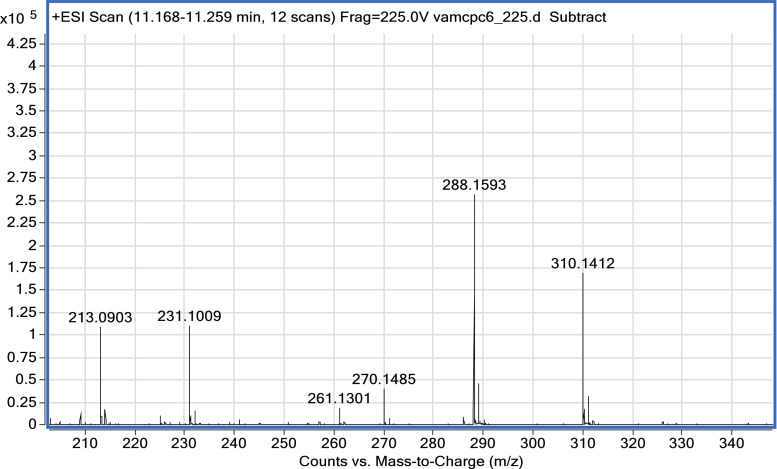
In-source ESI-TOF-MS of galanthamine obtained for the sample

This study revealed the presence of galanthamine in root extracts of Mexican poppy (*Argemone mexicana* L.). The compound was identified in fraction 6 and subsequently purified on an LH-20 Sephadex column in a solvent system MeOH–H_2_O (50:50), and yielded 2.95 mg. As far as we are aware, this is the first time galanthamine has been reported not only in the tissues of Mexican poppy, but also in the whole Papaveraceae family. Amaryllidaceae plants have been treated as the main sources of this pharmacologically precious alkaloid. In view of this finding, poppies might be perceived as alternative sources and be introduced into the treatment strategies for impaired cognition, because the percentage content of this alkaloid exceeded 0.77 % of the dried weight. According to Lopez et al. [26], the highest concentration of galanthamine in *Narcissus confusus* bulbs was calculated to be 2.5 % at most. The ESI-TOF-MS spectrum of galanthamine (Fig. 3) isolated from the root extract was compared with the similar spectrum of a reference solution. It was in agreement with ESI-TOF-MS data published elsewhere [24].

This is also the first report of the presence of palmatine and magnoflorine in extracts of prickly poppy.

### TLC bioautography of CCC fractions

TLC with fractions after CCC-based fractionation was directed to a TLC-bioautography by the optimized “Fast Blue B reagent” method [24]. The procedure was used to search for AChE inhibitors in *A. mexicana* DCM root extract. Galanthamine, a known inhibitor, was spotted on a TLC as a reference. Combining TLC-bioautography with TLC–HPLC–ESI-MS methods, it was possible to determine the structures of the active compounds.

Among the sprayed fractions, fraction 6 contained a vivid white spot indicating anticholinesterase properties. In fraction 26 there were two stronger and two weaker inhibitors.

**Fig. 4:**
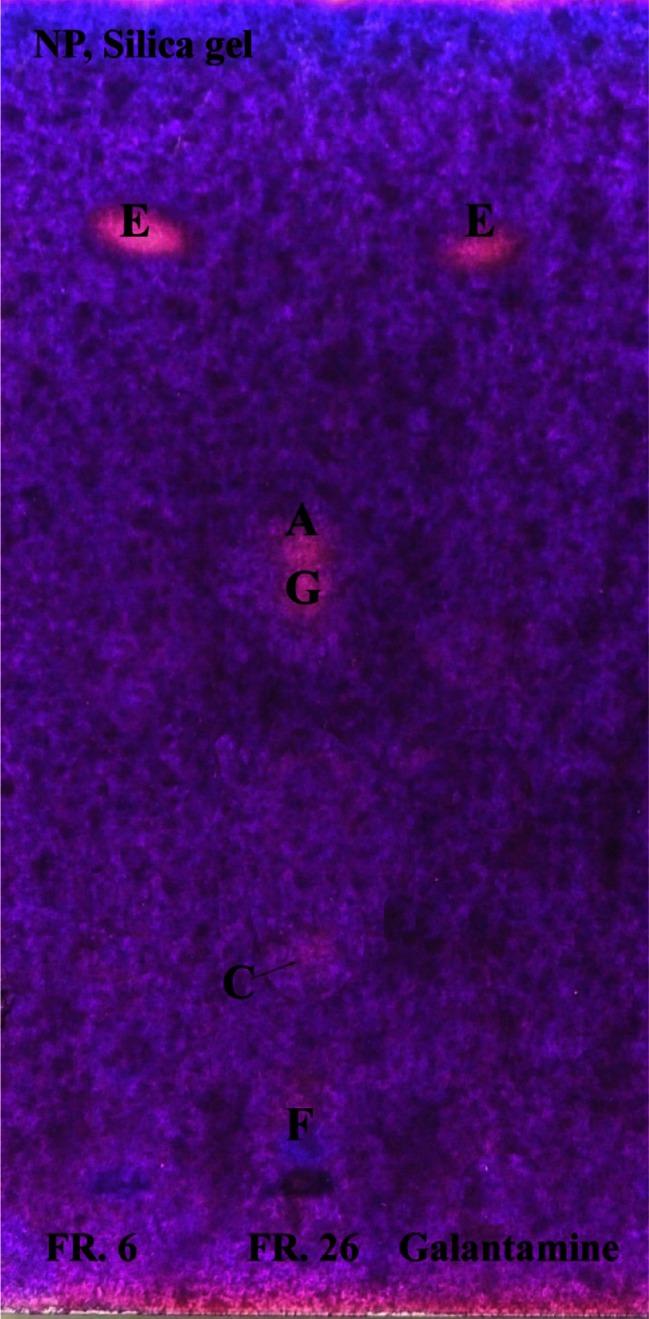
Results of TLC-bioautography from the “Fast Blue B” test on NP silica-gel TLC plates. Band 1 (fraction 6): inhibition zone of galanthamine in the upper third of the TLC; Band 2 (fraction 26): weak inhibition zones of (from the *top*) berberine, palmatine, protopine, magnoflorine; Band 3 (galanthamine): inhibition zone of galanthamine standard

Identification of active compounds was performed on a non-sprayed TLC to avoid the injection of a substrate added to the solvent mixture, and revealed the presence of galanthamine in fraction 6. Fraction 26 contained four active compounds which were identified as (counting from the top of track 26) berberine, palmatine, coptisine, and magnoflorine. A blueish, delicate band was present at the bottom of track 26, which might confirm weak anti-AChE properties of magnoflorine. Berberine and palmatine seem to be stronger inhibitors than the remaining compounds present in this fraction (Fig. 4). All compounds have been previously reported to be active inhibitors of acethylcholinesterase [27, 28].

These results reveal the CCC–TLC–HPLC–ESI-TOF-MS-based technique to be suitable for the screening of pharmacologically active natural products present in complex plant extracts. This rapid multistep technique enables quick recognition of anticholinesterase components with no need for their isolation. A “Fast Blue B reagent” assay was performed and resulted in the identification of the following inhibitors of AChE: galanthamine, berberine, palmatine, magnoflorine, and coptisine. Galanthamine (an alkaloid commonly used in AD therapy), palmatine, and magnoflorine were identified in Mexican prickly poppy for the first time, and this is the first time galanthamine has been identified in any plant of the Papaveraceae family.

## Abbreviations

AChE: Acetylcholinesterase
DCM: Dichloromethane
ESI: Electrospray ionization
HP-TLC: High-performance thin-layer chromatography plates
Hydrostatic CCC, CCC: Hydrostatic counter-current chromatography
MeCN: Acetonitrile
MeOH: Methanol
NH_3_: Ammonia solution
Rf: Retention factor
rpm: Rotations per minute
TLC: Thin-layer chromatography
TOF: Time-of-flight mass analyzer

## References

[1] Alzheimer’s Association (2014) 2014 Alzheimer’s disease facts and figures. Washington DC 10.1016/j.jalz.2014.02.001 24818261

[2] Schneider LS, Mangialasche F, Andreasen N, Feldman H, Giacobini E, Jones R, Mantua V, Mecocci P, Pani L, Winblad B, Kivipelto M (2014). Clinical trials and late-stage drug development for Alzheimer’s disease: an appraisal from 1984 to 2014. J Intern Med.

[3] Tomassoni D, Catalani A, Cinque C, di Tullio MA, Tayebati SK, Cadoni A, Nwankwo IE, Traini E, Amenta F (2012). Effects of cholinergic enhancing drugs on cholinergic transporters in the brain and peripheral blood lymphocytes of spontaneously hypertensive rats. Curr Alzheimers Res.

[4] Verma SK, Dev G, Tyagi AK, Goomber S, Jain GV (2001). Argemone mexicana poisoning: autopsy findings of two cases. Forensic Sci Int.

[5] Ghosh P, Reddy KMM, Sashidhar RB (2005). Quantitative evaluation of sanguinarine as an index of argemone oil adulteration in edible mustard oil by high performance thin layer chromatography. Food Chem.

[6] Singh S, Singh TD, Singh VP, Pandey VB (2009). Alkaloids of Argemone mexicana. J Indian Chem Soc.

[7] Singh S, Singh TD, Singh VP, Pandey VB (2010). Quaternary alkaloids of Argemone mexicana. Pharm Biol.

[8] Duke JA, Bogenshutz-Godwin MJ, Ottesen AR (2009). Duke’s Handbook of Medicinal Plants of Latin America.

[9] Hatfield G (2004). Encyclopedia of folk medicine: old world and new world traditions.

[10] Srivastava N, Sharma RK, Singh N, Sharma B (2012). Acetylcholinesterase from human erythrocytes membrane: a screen for evaluating the activity of some traditional plant extracts. Cell Mol Biol.

[11] Foucault AP, Chevolot LJ (1998). Counter – current chromatography: instrumentation, solvent selection and some recent applications to natural product purification. J Chromatogr A.

[12] Marston A, Hostettmann K (2006). Developments in the application of counter – current chromatography to plant analysis. J Chromatogr A.

[13] Ito Y, Ma Y (1996). pH-Zone-refining countercurrent chromatography. J Chromatogr A.

[14] Bourdat-Deschamps M, Herrenknecht C, Akendengue B, Laurens A, Hocquemiller R (2004). Separation of protoberberine quaternary alkaloids from a crude extract of Enantia chlorantha by centrifugal partition chromatography. J Chromatogr A.

[15] Zhang S, Wang M, Wang C (2011). Preparative separation and purification of alkaloids from Rhizoma coptidis by high-speed counter-current chromatography. Sep Purif Technol.

[16] Yang F, Zhang T, Zhang R, Ito Y (1998). Application of analytical and preparative high-speed counter-current chromatography for separation of alkaloids from Coptis chinensis Franch. J Chromatogr A.

[17] Berthod A, Hassoun M, Ruitz-Angel MJ (2005). Alkane effect in the Arizona liquid system used in counter current chromatography. Anal Biol Chem.

[18] Dahlmann J, Budakowski WR, Luckas B (2003). Liquid chromatography-electrospray ionisation-mass spectrometry based method for the simultaneous determination of algal and cyanobacterial toxins in phytoplankton from marine waters and lakes followed by tetative structural elucidation of microcystins. J Chromatogr A.

[19] Grace MH, Warlick CW, Neff SA, Lila MA (2014). Efficient preparative isolation and identification of walnut bioactive components using high-speed counter-current chromatography and LC-ESI-IT-TOF-MS. Food Chem.

[20] Chen J, Wang F, Liu J, Lee FSC, Wang X, Yang H (2008). Analysis of alkaloids in Coptis chinensis Franch by accelerated solvent extraction combined with ultra performance liquid chromatographic analysis with photodiode array and tandem mass spectrometry detections. Anal Chim Acta.

[21] Deevanhxay P, Suzuki M, Maeshibu N, Li H, Tanaka K, Hirose S (2009). Simultaneous characterization of quaternary alkaloids, 8-oxoprotoberberine alkaloids, and a steroid compounds in Conium fenestratum by liquid chromatography hybrid ion trap time-of-flight mass spectrometry. J Pharm Biomed Anal.

[22] Zhou Q, Liu Y, Wang X, Di X (2012). A sensitive and selective liquid chromatography-tandem mass spectrometry method for simultaneous determination of five isoquinoline alkaloids from Chelidonium maius L. in rat plasma and its application to a pharmacokinetic study. J Mass Spectrom.

[23] Qing Z-X, Cheng P, Liu X-B, Liu Y-S, Zeng J-G (2015). Systematic identification of alkaloids in Macleaya microcarpa fruits by liquid chromatography tandem mass spectrometry combined with the isoquinoline alkaloids biosynthetic pathway. J Pharm Biomed Anal.

[24] Mroczek T (2009). Highly efficient, selective and sensitive molecular screening of acetylcholinesterase inhibitors of natural origin by solid-phase extraction-liquid chromatography/electrospray ionisation-octopole-orthogonal acceleration time-of-flight-mass spectrometry and novel thin-layer chromatography-based bioautography. J Chromatogr A.

[25] Kukula-Koch W, Aligiannis N, Halabalaki M, Skaltsounis AL, Glowniak K, Kalpoutzakis E (2013). Influence of extraction procedures on phenolic content and antioxidant activity of Cretan barberry herb. Food Chem.

[26] Lopez S, Bastida J, Viladomat F, Codina C (2013). Galanthamine pattern in *Narcissus confusus* plants. Planta Med.

[27] Hu Y, Yang Y, Yang W, Zhang Y (2014). In vitro studies on the multi-target anti-Alzheimer activities of berberine-like alkaloids from Coptidis rhizoma. J Chin Pharm Sci.

[28] Yusoff M, Hamid H, Houghton P (2014). Anticholinesterase inhibitory activity of quaternary alkaloids from tiospora crispa. Molecules.

